# The aporetic dialogues of Modena on gender differences: Is it all about testosterone? EPISODE I: CRIME

**DOI:** 10.1111/andr.13797

**Published:** 2024-11-07

**Authors:** Giulia Brigante, Giulia D'Angelo, Vanessa Caccin, Silvia Coluccia, Immacolata Conte, Veronica Maria Demichelis, Rossana Cecchi, Manuela Simoni

**Affiliations:** ^1^ Department of Biomedical, Metabolic and Neural Sciences Unit of Endocrinology University of Modena and Reggio Emilia Modena Italy; ^2^ Department of Medical Specialties Unit of Endocrinology, Azienda Ospedaliero‐Universitaria of Modena Modena Italy; ^3^ Department of Biomedical, Metabolic and Neural Sciences Unit of Legal Medicine, University of Modena and Reggio Emilia Modena Italy

**Keywords:** aggressive behavior, crime, gender, testosterone

## Abstract

This is the first episode of a series of four discussions on the differences between males and females, in apparently non‐andrological fields. You will read the transcript of discussions that actually took place at the Endocrinology Unit in Modena, Italy, in the form of the aporetic dialogues of ancient Greece. In this episode, the role of testosterone in gender differences in criminal behavior will be explored. The discussants were divided into two groups: group 1, which supports the thesis of a predominant role of testosterone, and group 2, which opposes it. The first group affirmed that both endogenous testosterone and anabolic‐androgenic steroids could trigger aggressive and criminal behavior, regardless of predisposition to psychiatric disease or sociocultural background. The second group asserted the multifactorial genesis of aggressive and criminal behavior, citing other hormonal and non‐hormonal factors, such as neurotransmitters, cortisol, and sociological and psychological aspects. In the end, a forensic physician, acting as a referee, tried to resolve the aporia: are the two theories equivalent or one is superior?

## 1 INTRODUCTION

In recent years, there has been an increase in the publication of articles regarding gender differences. This derives from the awareness of clear differences between genders in many life aspects. However, numerous papers have been dedicated to the in‐depth study of biological phenomena in males and, only a few, in females. The vast majority of published studies concern purely biological aspects, from the prevalence of diseases to the effectiveness of treatments, etc. However, gender‐related differences are evident also in social and cultural phenomena.

In this article, the first of a series of four, we discuss the study of gender differences with a multidisciplinary approach, aiming to analyze the possible influence of the sexual hormone testosterone on behaviors and socio‐cultural variables. In particular, starting from the analysis of data on gender differences in committing criminal acts, the authors questioned the role of testosterone in determining these differences. On one hand, we considered the reasons that identify testosterone as the only or the main relevant factor in the genesis of aggressive phenotype, and on the other hand, we analyzed violent attitude as a multifactorial phenomenon, caused by other hormonal, social, cultural, and anthropological factors.

## 2 METHODOLOGY

The discussion was carried out according to the scheme of the Socratic aporetic dialogue: the question of the definition or explanation of a phenomenon is posed and then discussed, without necessarily reaching a truth. Aporetic dialogues typically end in *aporia*, a state of puzzlement by way of the equality of opposite reasonings. The discussants could discover that the opposing reasons only apparently balance each other and therefore identify the most promising theory, or that the two theses are actually equivalent, so the investigation must take a new turn or stop.

The discussants are subdivided into two groups: group 1 in favor of a prominent testosterone role, and group 2 against it. The dialogue took place as a seminar event open to the public.

Immacolata Conte (IC) presented objective data from official Italian national statistics on criminal activities carried out or suffered by men and women. Then, Silvia Coluccia (SC) and Vanessa Caccin (VC) tried to demonstrate that the evident gender differences in this context are due only to the effect of testosterone (group 1). On the other hand, Veronica Maria Demichelis (VMD) and Giulia D'Angelo (GDA) attempted to dismantle their thesis and demonstrate that other hormonal, social, cultural, and anthropological factors also come into play (group 2).

Rossana Cecchi (RC), the forensic physician, had the role of commenting on the data, functioning as a referee, and, in the end, deciding whether one of the two theories was successful or whether the two were equivalent.

The discussion was also open to questions or comments from the audience made up of endocrinologists and residents, biologists, biotechnologists from the Endocrinology Unit of Modena, residents from the School of Forensic Medicine, and from the School of Psychiatry (audience).

The organization of the dialogue, the literature search, and the collection of the data presented were managed and supervised by Giulia Brigante (GB), based on an idea by Manuela Simoni (MS).

## 3 THE APORETIC DIALOGUE

**IC**: First of all, we need to understand if there is a gender difference in the way of committing criminal acts. That's why I am going to show you some epidemiological data from the official Italian National Institute of Statistics (ISTAT).

Analyzing ISTAT data from the year 2022, there is a clear gender difference among Italian prisoners: 29,102 were males versus 1048 females. This remains true if different kinds of crime are analyzed separately: males are responsible for 97% of voluntary murders, for 93% of manslaughter, for 98% of negligent murder, for 98% of voluntary personal injury, for 96% of negligent personal injury, for 97% of private violence and threat, for 98% of domestic violence, for 98% of sexual violence, for 98% of beatings, and 97% of other kind of crimes against the person.^1^, ^2^ Therefore, the preponderance of the male gender in crime is objectifiable and undeniable. Overall, 94% of all murders were committed by males, confirming a clear gender polarization.

If we consider murder victims, out of a total of 322, 39% were females and 61% males. Interestingly, the vast majority of male victims (94.4%) were killed by males, as well as 93% of female victims.^1^, ^2^ Male victims were mainly aged 35–45 years, while female victims have a first peak at 35–44 years, then incidence tends to increase as the age of victim increases.^1^, ^2^

Regarding the victim‐killer relationship, 55% of male victims did not know the murderer, while 49% of female victims had an intimate relationship with the killer.^1^, ^2^

The main murder motives are arguments, futile reasons, and grudges (33% against female victims, 53% against male victims), followed by economic reasons (8% against females, 18% against males), state of “insanity” of the perpetrator (18% against females, 7% against males), passionate reasons (17% against females, 5% against males), desire to stop victim's suffering (4% against females, 0% against males). The last two reasons are mainly present in crimes committed against female victims.^1^, ^2^

**RC (Referee)**: It is interesting to note that “mercy killings”, aimed at ending a victim's suffering, predominantly involve female victims and are often perpetrated by males. A possible explanation could be that males have a lower tolerance for the suffering of others, leading them to intervene forcefully. In contrast, females may exhibit greater tolerance and support for the sufferer until the end.

**IC**: Regarding murder methods, males are more frequently victims of firearms (24% of female victims, 45% for male victims), whereas women are more likely to be killed by methods involving direct physical contact such as bare hands (30% of female victims, 15% for male victims), implicating body‐to‐body contact and more aggression and anger towards the victim, edged weapon (35% of female victims, 31% for male victims) and blunt instrument (11% of female victims, 8% for male victims).^1^, ^2^

In conclusion, analysis of ISTAT data reveals a significant gender disparity in criminal attitude in terms of crime numbers, methods of murder implementation, and victim‐perpetrator relationship. There is a notably higher percentage of male perpetrators who exhibit greater aggression and cruelty. Moreover, females are predominantly murdered in domestic settings, often by a current or former male partner.

Now, we will delve deeper into the potential role of testosterone in influencing these gender differences.

**SC (group 1)**: Certainly, not all men are inherently violent, aggressive, or ruthless murderers. However, it is well‐documented that a significant proportion of violent crimes are committed by men, leading many researchers to affirm that testosterone plays a key role in aggression. I agree with this theory and I will present some studies demonstrating the link between high testosterone and ruthless crimes. In the first one, the authors measured salivary testosterone levels in 230 male inmates convicted of violent crimes (including murder, voluntary manslaughter, robbery, armed robbery, assault, rape, and child molestation).^3^ They examined the personal files of the ten inmates with the highest testosterone levels and the ten with the lowest levels, finding a greater propensity for ruthlessness among inmates with higher testosterone levels. One very violent case concerns a killer who shot his sister through the door with a rifle, probably killing her instantly, then entered the room and shot her in the stomach. Then shot her twice in the head with a pistol and cut her throat nearly from ear to ear. In another case that was violent and especially coldblooded, one victim was hit in the head twice with a lead pipe and then shot in the head. Another victim was shot twice in the head. While waiting for the victims to die, the perpetrators sat in another room and smoked some pot.^3^ Actually, the most heinous crimes were committed by men with higher testosterone levels.

Furthermore, beyond endogenous testosterone, we must consider anabolic‐androgenic steroids (AAS) too. Non‐medical use of AAS has emerged as a worldwide public health issue.^4^ It is now generally accepted that some AAS users develop violent or criminal behaviors during assumption.^5^ This could be due to direct biological effects or triggering of predisposed psychopathologies in certain socio‐cultural contexts.^5^

To convince you, I will provide several clinical case examples of steroid abusers who committed violent criminal acts.

Case 1^5^: Mr. A, was a 58‐year‐old man, who was a victim of sexual abuse at the age of 11. At age 15 he first used AAS, and then started using them chronically from the age of 20 with the aim to maintain good physical condition and to achieve more success in the stripper industry. After ending his career at 56, he took on a job as a bouncer, using consistent doses of AAS. He reported feeling “invincible”. Being a supporter of Donald Trump, he claimed to have become obsessed with the prejudices of left‐wing persons. He decided to do something to scare or discourage prominent persons, as a result, created crude homemade devices, and bombs, and sent them to important public figures, including Barack Obama, Hillary Clinton, George Soros, and Robert De Niro. Mr. A was swiftly arrested and imprisoned. With the abrupt cessation of AAS, his feelings of invincibility vanished and he looked back in disbelief at what he had done.

Case 2^5^: Mr. B was an aspiring young athlete going to secondary school with a very successful football program when he first used AAS. In a forensic psychiatric interview, he reported that his most serious crime prior to AAS use was stealing a pack of chewing gum from a convenience store. He reported no prior history of any psychiatric disorder, nor of violent or aggressive behavior. During steroid use, he felt ‘invincible’. He desired to make an expensive purchase to impress his girlfriend. To get the money, he resolved to break into a seemingly unoccupied house to steal valuables. He opened a window with a hunting knife, began searching the house, and was shocked when the owner suddenly appeared, confronted him, and attempted to restrain him. He stabbed the owner nine times with the hunting knife, concluding by slitting his throat. He then heard the owner's wife in a neighboring room attempting to call the police and stabbed her multiple times, again concluding by slitting her throat. Once incarcerated and no longer exposed to AAS, he promptly reverted to his original benign personality. He reported feeling deep remorse for his actions and being unable to comprehend how he could have committed such a crime.

In both cited cases, the killers obtained reduced sentences since crimes were attributed to the use of substances able to alter the subject's mental capacity.^5^

Case 3 (6): a 32‐year‐old male went to the police to confess to having just killed his girlfriend by inflicting several stabs with a kitchen knife. He was very nervous and particularly aggressive. About 90 min after the assault, a blood specimen was collected and it was free of alcohol, pharmaceuticals, and drugs of abuse, but tested positive by liquid chromatography‐tandem mass spectrometry for methandienone (32 ng/mL) and trenbolone (9 ng/mL). The perpetrator admitted to regular consumption of anabolic steroids to enhance his muscular mass, as he was a professional security agent. These concentrations are highly indicative that the murderer was under the influence of anabolic steroids at the time of the assault.

All the above‐mentioned cases suggest an association between misuse of anabolic steroids and irritability/aggression. Abusers of steroids are more prone to be involved in criminal acts, as demonstrated also by epidemiological studies.^7^, ^8^, ^9^, ^10^

**RC (referee)**: An important point to consider is that committing a crime under the influence of substances that alter mental faculties, such as drugs or alcohol, may influence the offender's responsibility for their actions, or even render them not responsible at all when the abuse is chronic and the mental faculties are constantly altered. So, we must be very cautious in attributing aggression solely to the use of anabolic steroids, which alter behavior. There is no such strong evidence, from randomized clinical trials, that demonstrates that anabolics alter mental faculties making the subject incapable of understanding and will. It is possible that the subjects of the cases described above had a predisposing psychological background. For example, for case 1 it is specified that he was a victim of sexual abuse in childhood.

**VMD (group 2)**: So maybe it's not only testosterone's fault if men commit crimes more and more violently than women. I aim to demonstrate that a multitude of factors other than testosterone are involved in criminal attitude.

Neurotransmitters such as serotonin, dopamine, and noradrenaline are involved in modulating impulsive‐aggressive behaviors.^11^ The brain serotoninergic system is considered one of the main regulators of proactive aggression: reduced levels of serotonin or its metabolite, 5‐hydroxyindoleacetic acid, in cerebrospinal fluid are correlated with increased aggression and antisocial personality disorder.^12^

Studies on murine models provide further insights. For instance, mice knockout for the *TPH2* gene, which encodes the tryptophan hydroxylase type 2 (TPH2), an enzyme essential for serotonin synthesis, exhibit minimal serotonin expression in the brain, correlating with a significant increase in aggression, assessed as latency to respond to attack, quantity, and duration of attacks.^13^

In humans, mutations in genes encoding proteins involved in serotoninergic system regulation are associated with more aggressive behaviors. Analysis of six polymorphisms in genes implicated in the serotoninergic system was conducted on 370 convicted murderers in Pakistani prisons compared to 359 non‐violent men highlighted a prevalence of mutations in the prisoners, suggesting that mutations in these genes may be correlated with the development of criminal behavior.^13^

The serotoninergic system is also modulated by the environment. Mice exposed to offspring or cohabitating with a companion mouse had a lower propensity for aggression towards pups, despite reduced concentrations of brain serotonin.^14^ Adverse events or exposure to particular risk factors during the developmental period can alter the serotoninergic response to stress and cause a disruption of the normal nervous system development, thus paving the way for future aggression.^15^

**SC (group 1)**: Right, neurotransmitters play a role in aggression but do not explain the gender difference.

**VMD (group 2)**: A gender difference has also been demonstrated in neurotransmitters, although there are few studies on the subject.^16^, ^17^, ^18^, ^19^, ^20^, ^21^, ^22^, ^23^, ^24^, ^25^, ^26^, ^27^ Interestingly, sex differences in 5‐HT regulation of aggression have been shown.

In 2008, Soloff et al. investigated the relationships of gender, impulsivity, aggression, and temperament to 5HT2A receptor binding in 21 healthy subjects using [^18^F]altanserin and PET neuroimaging. The study found significant gender differences, with males showing higher binding potential values than females, suggesting a greater number or affinity of 5HT2A receptors in certain brain regions for males.^26^ Additionally, research on Syrian hamsters showed that hypothalamic injections of 5‐HT1A agonists increased aggression in females but decreased it in males. Activation of 5‐HT neurons in the Dorsal Raphe Nucleus was linked to dominant behavior, but only in female hamsters.^27^

Another very interesting factor in this context of gender difference is the monoamine oxidase A (MAOA) gene. It encodes the MAOA enzyme, which metabolizes neurotransmitters such as serotonin, dopamine, and norepinephrine.^28^, ^29^ Congenital MAOA deficiency, known as the ‘warrior gene’,^28^ as well as low‐activity MAOA variants, have been associated with a higher risk of antisocial behavior and violence, as reported in several studies.^29^, ^30^

The MAOA gene is located on the X chromosome; therefore, males affected by these variants have only one copy and produce no MAOA enzyme.^29^

Thus, in men, the impact of MAOA variants is more pronounced, explaining the multifactorial nature of aggressive behavior in this gender.

Now, I would focus on some neuroanatomical aspects possibly involved in criminal attitude. Anomalies such as reduction in grey matter volume in the amygdala, hippocampus, and nucleus accumbens, are associated with psychopathic traits.^31^, ^32^

Therefore, it has been questioned whether brain anatomical differences are correlated with a more aggressive attitude.

I would like to present a recent study investigating the correlation between agonistic aggression and brain structure alterations in 22 female judo athletes compared with 22 healthy non‐athlete women. Both groups were subjected to questionnaires to assess aggression, the presence of antisocial aspects, and empathy, and all underwent magnetic resonance imaging (MRI) scans to calculate gray matter volume. It emerged that athletes were more prone to adopt aggressive and antisocial attitudes while scoring lower on empathy questionnaires. From MRI images, a significant reduction in gray matter volume was observed in the left occipital cortex, right temporal pole, and left amygdala in athletes. Negative correlations were observed between amygdala and hippocampus gray matter volumes and aggression scores.^33^

Normally, men have larger volumes and densities in the left hemisphere, while women have a more developed right hemisphere. The amygdala normally has larger dimensions in men, while the hippocampus is usually larger in women, permitting them to remember the smallest details of emotional experiences.^34^ Gender differences in amygdala function are notable: the female amygdala is more often activated by emotional nuances, while the male amygdala mainly maintains its ancestral functions, it registers fears and triggers aggressiveness. This could explain why women tend to respond emotionally to stress, anger, or fear, while men often engage the prefrontal cortex for motor‐oriented responses involving physical action.^35^ These behavioral differences can also be understood from an evolutionary perspective: in the face of danger, women instinctively think first of saving their children and try to placate conflicts, while men's role is to attack and drive away the aggressor.^36^

So, I believe that aggressive behavior involves multifactorial elements.

**Audience**: We have to pay attention to consider someone dangerous only because of certain physical characteristics, including neuroanatomical traits. There is a risk of falling into the so‐called “born criminal” theory formulated by Cesare Lombroso in the 19th century. He claimed that criminals could be identified through general characteristics, which he designated as composing a criminal type.^37^ Although his theories have been considered scientifically controversial, Lombroso himself is also credited with attempting to apply a clinical‐therapeutic approach in criminology.

**GB**: I agree, it's risky. I would like to add that, except for the latest study on female athletes, the majority of studies involve almost exclusively males. The gender gap is widespread in research, particularly where it is insurmountable since studies solely on women are rare, and the comparison between males and females is difficult.

**RC (referee)**: Studies on aggressive behaviors in women encounter reluctance among researchers. It is a cultural issue: we need to get used that women could be violent and exhibit “masculine” behaviors.^38^

**Audience**: It is difficult to accept that a woman can commit violent acts. We tend to reject it and not want to deal with it. We usually consider testosterone as a sex hormone, involved in sexuality. This correlation testosterone–sex is even much more direct in animals. Indeed, other features like cognitive aspects, social and behavioral contexts, religious inhibitions, etc must also be considered in humans. For example, some researchers demonstrated that if we block aromatization, and thus estrogen production in animals, there is a change in mate choice and sexual behavior, often leading to homosexual behavior.^39^ This is not evident in humans where also cognitive aspects must be considered. Indeed, individuals with a congenital absence of aromatase, which means incapable of converting testosterone into estradiol, do not exhibit homosexual sexual behavior.^40^

Thus, it is difficult to draw parallels between animals and humans in terms of sexuality, and even more so about criminal behavior.

**VC (group 1)**: VMD has just explained some neuroanatomy topics related to gender differences in aggression. Regarding the size of the right amygdala, it seems that its enlargement may be determined by the chronic intake of anabolic steroids too, so let's bring the focus back to testosterone.

Chronic intake of anabolic steroids seems to induce an increase in right amygdala volume, as well.

In 2015, a group of psychiatrists recruited 20 weightlifters. Half of them were chronic users of anabolic steroids. Using functional MRI (fMRI) and resting state fMRI (rs‐fMRI), the authors assessed the volume of the right amygdala and its various connections with other areas of the brain. Interestingly, the right amygdala in those who chronically used anabolic steroids was significantly larger, and its connections with other brain areas were reduced.^41^ This work suggests that anabolic steroids play a role in the central nervous system, although the way they actually act and influence behavior is still a subject of study.

Now I will show the results of a study that I consider particularly interesting regarding the role of testosterone in aggressiveness. In 1993, 20 healthy volunteers, who were not taking medication and had no comorbidities, were recruited and given increasing doses of methyl‐testosterone from day 1 to day 12. They were required to fill out a diary every day and self‐administer validated questionnaires. Then, they were asked to fill out other questionnaires administered by the nursing staff. Scores related to symptoms such as irritability and aggressiveness increased on days when subjects took the highest dose of methyl‐testosterone.^42^

In another study, 47 participants were divided into two groups, acting alternatively as cases and controls, and were administered testosterone cypionate at increasing doses from week 1 to week 6. During treatment, enrolled subjects had to complete a validated questionnaire (Young Mania Rating Scale) and they underwent a computer test (PSAP) with the aim of earning money by challenging the computer itself. The scores obtained on the questionnaire and PSAP test were higher during treatment with higher doses of testosterone.^43^

In Turin, 52 transgender patients who were starting the transition process from female to male, completed a questionnaire (STAXI‐2) at baseline, before starting testosterone treatment, and 1 and 7 months after starting treatment. A threshold of 75 was established to determine a normal level of aggressiveness. It was observed that at baseline when no one was yet taking testosterone, most participants had a score below 75, while after 7 months of treatment, the score obtained by most patients was above 75.^44^

Finally, I will present the results of a study aimed at analyzing the relationship between AAS and violent behavior. The Anti‐Doping Denmark, a public independent institution promoting the fight against doping in sport, established a collaboration with Danish commercial gyms and fitness centers to initiate a doping control program. Participants suspected of anabolic steroid abuse based on physical appearance underwent urine tests. From 2006 to 2018, they sanctioned 1219 people and 545 participants were recruited and checked in the Danish Central Crime Register to see if participants at the time of sanction had already a previous sentence. They compared data obtained with a sample from the general population and found a higher percentage of convicted individuals than those taking anabolic steroids. They conducted a multivariate analysis, considering possible confounding factors, like psychiatric comorbidities, medication use, alcohol and drug consumption, occupational status, and level of education, and the difference between the two groups remained significant, highlighting a predominant role of testosterone in determining aggressiveness and violent behavior.^45^

**Audience**: Until now, we have talked about subjects with high testosterone levels. What about anti‐estrogen users? They have high testosterone and low estrogen levels. They could be a model to understand if estrogen also plays a role in determining gender differences in crime.

**MS**: And what about the role of estrogens per se? Indeed, nowadays we have no doubt that testosterone provokes aggressiveness. For example, Berthold's experiment in which roosters became calmer after castration. However, we should also investigate how estrogens influence behavior. Another observation is that women feel stronger and invincible during pregnancy, as much as men using anabolic steroids.

**GDA (group 2)**: VC and SC, you have presented interesting papers about the key role of testosterone in criminal and aggressive attitudes. But maybe they have some limits because they do not account for other hormones that could modulate the effect of testosterone on aggressive behavior.

A very interesting issue is the dual hormone hypothesis (DHH), which proposes an association between testosterone and aggressive behavior modulated by cortisol serum levels.^46^ Cortisol is positively associated with fear, punishment sensitivity, anxiety, and social avoidance; this therefore suggests that low cortisol/decreased stress system activity may be associated with hypo‐arousal and increased antisocial behavior. According to this theory, aggressive behaviors occur more frequently when testosterone levels are high and cortisol levels are low.

Below, I will report some papers supporting the DHH.

The first one investigated the possible association between testosterone and cortisol salivary levels, in samples collected via passive drool, and self‐report aggression using the Reactive Proactive Aggression Questionnaire. A total of 862 undergraduate university students were enrolled, 366 females and 186 males of average age of 20 years. Interestingly, cortisol levels were significantly lower in those who had higher scores on the questionnaire, which means those with the greatest aggressiveness, especially among women. In contrast, baseline testosterone and change in testosterone were not correlated with any of the aggressive behavior measures.^47^

The same author published another study investigating the relationship between cortisol and testosterone and criminal behavior, evaluated by a set of 38 self‐report items. Data were gathered from a sample of 552 undergraduate students, revealing a statistically significant positive effect of testosterone on crime when cortisol levels were below 0.70 g/dL under the mean, especially in males.^48^

These are just two examples of the numerous studies on the topic that clearly demonstrate that cortisol plays an important role in modulating the effect of testosterone on aggressiveness. In this context, anything that alters cortisol levels has a cascading effect. For example, the stress of childhood abuse is associated with adverse influences on brain development and the neuroendocrine production of cortisol.^49^, ^50^

The following study examined the relationships between basal resting salivary cortisol levels, neurologic abnormalities identified during physical examination, histories of physical or sexual abuse, and possible negative interferences on neurologic and brain development, with violent crime. A total of 113 female prison inmates, 27 convicted of violent crimes and 86 convicted of nonviolent crimes, were enrolled. Both those incarcerated for violent and nonviolent crimes had lower mean morning cortisol levels compared to adult females. Those currently incarcerated for a violent crime had significantly lower morning cortisol levels than those incarcerated for a nonviolent crime. 59% of all subjects had abnormal neurologic histories and/or neurologic examination abnormalities, probably as a result of childhood abuse and more recent abuse. The authors suggest that low cortisol morning levels could be the result of damage to the hypothalamus that may have resulted from physical or psychological trauma.^51^

Regarding gender differences in the effects of cortisol and specifically the DHH on aggression, there is, to our knowledge, no clear evidence in the literature.

However, women typically have more self‐control than men and a more efficient emotion‐regulation system.^52^ Therefore, when females experience high levels of HPA axis activity, it seems to impair normally strong inhibitory processes, leading to reduced inhibition and resulting in a level of aggressive behavior comparable to that of men.^53^, ^54^, ^55^ As Böhnke et al. demonstrated in their study, in which they administered hydrocortisone to their patients to increase acute circulating cortisol levels. Women generally became more aggressive, displaying a level of aggressive behavior comparable to that of men. In contrast, men did not show a significant change in behavior.^53^

Moreover, under stressed conditions, females seem to have a better‐functioning HPA axis, which leads to reduced aggressiveness.^56^ However, in conditions where the HPA axis is impaired or altered, women also exhibit aggressive behavior similar to that of men.

**MS**: The presented studies were based on cortisol and testosterone measurements from saliva samples, but it is important to underline that these are not the gold‐standard measurement methods.

**GDA (group 2)**: Yes, that is true, and steroid hormone measurement from saliva samples is based on immunological methods (radioimmunoassay, enzyme immunoassay, or enzyme‐linked immunosorbent assay), while mass spectrometry is the gold standard. However, saliva collection is less invasive than blood sampling.^57^ This is the reason why this method is preferred in many studies, especially if conducted by researchers who are not endocrinologists.

**Audience**: People with personality disorders are often more prone to commit crimes. So, why don't we measure their cortisol and/or testosterone serum levels, to verify if they are within normal ranges?

**GDA (group 2)**: I think there is an important matter here. If we find cortisol or testosterone levels deviating from mean, but not reaching pathological levels, what shall we do? Should we treat them to “prevent” a possible crime? Would it be ethical? Would this be medically justified?

To conclude, I want to leave you some food for thought from human sciences to try to answer the question: why do men commit more crimes than women?

I will bring back the theory of precarious manhood, according to which the man of any culture has a continuous need to show his manhood to his peers. However, the concept of manhood is closely associated with the concept of aggressiveness, as explained in an essay from 1988, reporting the example of an Amazonian population where men who married more women were also those who committed more killings.^58^, ^59^

But then we wondered why especially young men commit crimes. To answer this question, I will tell you something about the “Young Male Syndrome”, an essay that analyzes the probability of dying from murder at various stages of life, distinguishing males and females. It turned out that there are no gender‐related differences in the odds of dying of murder in childhood and in the elderly. Differently from the Italian data presented by IC at the beginning, this theory suggests that the main differences are in the age between 20 and 30 years, in which males have a greater probability of being killed. One possible explanation for this phenomenon could be that young men need to assert their manhood within society, even at the cost of committing or suffering violent actions.^60^

Finally, there is another interesting question: why do women commit fewer crimes?

Criminologist Freda Adler supports a very original and highly provocative thesis: women commit fewer crimes because they have not yet reached a level of emancipation that would make them equal to men. So only when they are fully emancipated, they will commit an equal number of crimes as men. She therefore describes a concept of emancipation as masculinization.^61^

This theory has been partially overcome, and today the most accepted theory is that women commit fewer crimes because they are subject to both social and formal control, whereas men only to formal control. Social control is exerted by families and social groups that impose on women a submitted role in regard to men, while formal control refers to legal and institutional oversight. So female gender results in over‐controlled respect to males, and in this prospect criminal behavior is a freedom manifestation, that women could not experience.^62^

**RC (referee)**: In the end, you are all right, since the basis of aggressiveness is multifactorial, including hormones such as testosterone and cortisol, as well as positive or negative life experiences. Maybe previous experiences may have an epigenetic role in biological mechanisms, predisposing the subject to hormonal and non‐hormonal influences which lead to greater aggressiveness. I think, for example, of the many cases cited in which the criminal had experienced previous histories of abuse.

**GB**: Perhaps males are less capable of overcoming these traumas and this makes them more mentally but also biologically predisposed to violent reactions later over time.

**RC (referee)**: Yes, it could be the case. I was struck by the case report about the man abusing anabolic steroids who had a history of childhood abuse. Another example: newborns from pregnant women during the period of the Twin Towers attack had higher cortisol levels, yet they were calmer.

In the forensic medical field, epigenetics is potentially dangerous since it could make someone not chargeable for his/her crimes.^63^

**GB**: Public opinion often tends to point to criminals as psychiatric patients. In reality, the majority of crimes, even the most heinous, are committed by people who are perfectly capable of understanding and wanting. Perhaps categorizing them as psychiatric might be a defense mechanism to make what seems unacceptable more comprehensible to us. There is another gender difference: we tend to see the male criminal as “bad” while the female criminal as “crazy”. Perhaps this is because it is more difficult to accept that females can also commit violent acts. A typical example is infanticide, a mother who kills her child. We struggle to accept that this can happen outside of psychiatric illness because we do not consider it acceptable.

**RC (referee)**: Yes, I agree with you. Talking about psychiatric disease and how it is hard to accept violence, we analyzed a wide series of feminicides and we found that psychiatric disease is not relevant.^64^ The killer is conscious about what is doing when committing the crime. I think that psychiatric disease is a way to shift responsibilities away from society. This refusal to face and understand violence is even more evident if the criminal is a female. This severely limits research studying crimes committed by women, creating a further gender gap.

**SC (group 1)**: I agree, but I think it's important to note that criminals often have an altered perception of emotions. In fact, a higher percentage of alexithymia has been observed in criminals compared to the general population.^65^ Alexithymia is a personality trait characterized by difficulty in identifying and communicating emotions, which has been found to be more common in men and is also associated with hypersexual behavior.^66^, ^67^, ^68^ It makes me think that testosterone might also have an effect on this aspect.

## 4 APORETIC CONCLUSION

In conclusion, the nature of aggressive behavior concerns multifactorial aspects, including the association with hormones, but it is not limited only to them. Several data show how the male gender is more prone to commit crime and in general to be more aggressive than women, inducing growing interest in the role of testosterone. Both endogenous testosterone and anabolic‐androgenic steroids could trigger aggressive and criminal conduct, regardless of predisposition for psychiatric disease or sociocultural background. But we have not to forget how personality and behavior derive from a complex neuronal system where numerous neurotransmitters are involved. The brain serotoninergic system is considered one of the main regulators of proactive aggression, which seems to be increased with reduced serotonin levels. Moreover, certain neuroanatomical anomalies are associated with psychopathic traits. Concerning the endocrine system, testosterone is not the only hormone influencing the psychological aspect and personality, and its effect is probably modulated by other hormones, such as cortisol. Cortisol is positively associated with fear, punishment sensitivity, anxiety, and social avoidance, and low levels may be associated with increased antisocial behavior (DHH).

Finally, sociologists and psychologists have focused on gender differences in crime. Across cultures, man typically needs to show aggressiveness as an expression of his manhood, especially during reproductive age. But also, woman conditions influence that attitude. According to criminologist Freda Adler, women commit fewer crimes because they have not achieved full emancipation. A more recent theory finds the reason in social and formal control: women are subject to both, whereas men primarily face formal control.

**FIGURE 1:**
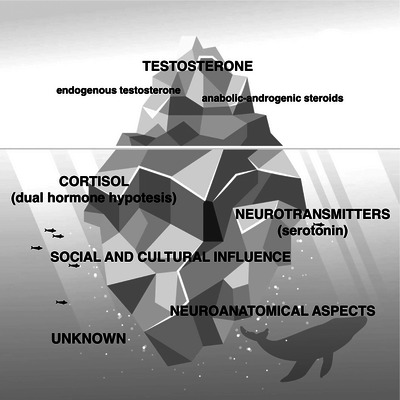
Do men and women commit crimes differently because of testosterone? Testosterone is the tip of the iceberg including all the factors that influence this gender difference.

In the end, referee Prof. Rossana Cecchi concluded that we are all right since criminal and aggressive behavior has a multifactorial nature, where hormones, neurotransmitters, and sociological and psychological aspects all play an important role in its genesis (figure 1).

## AUTHOR CONTRIBUTIONS

Manuela Simoni had the idea of the discussion on this topic and the aporetic dialogue structure; Giulia D'Angelo, Immacolata Conte, Silvia Coluccia, Vanessa Caccin, and Veronica Maria Demichelis did the bibliographic research and created the data presentation under Giulia Brigante's supervision; Rossana Cecchi, Manuela Simoni, Giulia Brigante, Giulia D'Angelo, Immacolata Conte, Silvia Coluccia, Vanessa Caccin, and Veronica Maria Demichelis discussed and commented on the data, wrote the paper; Giulia Brigante and Giulia D'Angelo submitted the research.

## CONFLICT OF INTEREST STATEMENT

The authors declare no conflicts of interest.

## FUNDING INFORMATION

This project has not received any funding.

## Data Availability

Data sharing is not applicable to this article as no new data were created or analyzed in this study.
